# Dissemination of endometrial cancer MRI staging guidelines among young radiologists: an ESUR Junior Network survey

**DOI:** 10.1186/s13244-023-01491-w

**Published:** 2023-09-04

**Authors:** Arnaldo Stanzione, Eduardo Alvarez Hornia, Ana Sofia Linhares Moreira, Luca Russo, Pamela Causa Andrieu, Carlos Carnelli, Renato Cuocolo, Giorgio Brembilla, Jeries Paolo Zawaideh, for the ESUR Junior Network Committee, Francesco Alessandrino, Nathania Bonanno, Vlad Bura, Maarten de Rooij, Marianna Konidari, Camilla Panico, Viktoria Palm, Martina Pecoraro, Andrea Ponsiglione, Doaa E. Sharaf, Carmelo Sofia, Chen-Jiang Wu

**Affiliations:** 1 Department of Advanced Biomedical Sciences, University of Naples Federico II, Naples, Italy; 2 Department of Radiology, Clinic IMQ Zorrotzaurre, Bilbao, Spain; 3 Radiology Department, Centro Hospitalar Universitário do Algarve, Faro, Portugal; 4 Fondazione Policlinico Universitario A. Gemelli IRCCS, Rome, Italy; 5 Memorial Sloan Kettering Cancer Center, New York, NY USA; 6 Hospital de Clínicas, Universidad de la República, Montevideo, Uruguay; 7 Department of Medicine, Surgery and Dentistry, University of Salerno, Baronissi, Italy; 8 Department of Radiology, IRCCS San Raffaele Scientific Institute, Vita-Salute San Raffaele University, Milan, Italy; 9 Department of Radiology, IRCCS Policlinico San Martino, Genova, Italy

**Keywords:** Endometrial cancer, Staging, MRI, Radiology trainee, Guidelines

## Abstract

**Objectives:**

Imaging guidelines could play an important role in the training of radiologists, but the extent of their adoption in residency programs is unclear. With this survey, the European Society of Urogenital Radiology (ESUR) Junior Network aimed to assess the dissemination of the ESUR guidelines on endometrial cancer MRI staging (EC-ESUR guidelines) among young radiologists.

**Methods:**

An online questionnaire targeted to last year radiology residents and radiologists in the first year of their career was designed. It included 24 questions, structured in 4 sections (i.e., background, general, acquisition protocol, interpretation, and reporting). The survey was active between April and May 2022, accepting answers worldwide. Answers were solicited with a social media campaign and with the support of national scientific societies. Subgroup analysis was performed based on variables such as subspecialty of interest and number of EC-ESUR guidelines consultations using the Wilcoxon rank sum test.

**Results:**

In total, 118 participants completed the questionnaire, of which 94 (80%) were from Europe and 46 (39%) with a special interest in urogenital radiology. Overall, 68 (58%) stated that the guidelines were not part of their residency teaching programs while 32 (27%) had never even consulted the guidelines. Interest in urogenital radiology as a subspecialty and EC-ESUR guidelines consultations were associated with greater confidence in supervising scan acquisition, interpreting, and reporting EC MRI staging exams.

**Conclusion:**

Four years after publication, the adoption of EC-ESUR guidelines in residency programs is heterogeneously low. Despite a possible selection bias, our findings indicate that active promotion of EC-ESUR guidelines is required.

**Key points:**

• The adoption of ESUR guidelines on endometrial cancer in radiology residency programs is heterogeneous.

• Almost one third of respondents stated they had never even consulted the guidelines.

• Confidence toward guidelines was higher in those who were exposed to more endometrial cancer MRI staging scans.

• Reading the guidelines was associated with a greater confidence in protocol acquisition, interpretation, and reporting.

• Active efforts to promote their dissemination are required.

**Supplementary Information:**

The online version contains supplementary material available at 10.1186/s13244-023-01491-w.

## Introduction

Imaging guidelines are intended to promote standardization and increase the quality of radiologists’ work, providing recommendations regarding clinical indications, acquisition protocol, image interpretation, and reporting. The number of available imaging guidelines has been steadily increasing, with most coming from collaborative working groups endorsed by scientific societies [1]. Great efforts have been put toward guideline development by the scientific community as their potential role in the professional training of future radiologists gained recognition [2, 3]. This notwithstanding, adherence to guideline recommendations is often poor and the lack of awareness or familiarity has been identified among the main barriers to a more widespread adoption [4].

Endometrial cancer is the fourth most common malignancy in females and imaging plays a pivotal role in the management of this disease, with MRI being considered the most accurate technique for local staging due to its excellent soft tissue contrast resolution [5, 6]. The European Society of Urogenital Radiology (ESUR) released an updated version of the endometrial cancer MRI staging guidelines (EC-ESUR guidelines) in mid-2018 [7]. Recently, the results of a 2019 survey have been published, suggesting that the use of ESUR guidelines on female pelvis MRI is less common among young radiologists compared to senior ones [8]. In this light, the main purpose of our study was to assess the dissemination extent of EC-ESUR guidelines as well as to explore their potential and perceived educational value among last year radiology trainees and young radiologists. The secondary aim was to obtain insights on how to increase their adoption in this group.

## Materials and methods

### Target population and questionnaire structure

A web-based survey was designed using Google Form (Google Inc., CA, USA). The target survey population was defined as radiology trainees in the last year of residency and radiologists within their first year of practice after residency. A questionnaire was drafted, revised, and finalized when all authors reached a consensus about its content. In total, it included 24 questions structured in 4 sections: “background” (4 questions), “general” (6 questions), “acquisition protocol” (6 questions), and “interpretation and reporting” (6 questions). Additionally, a question was asked before participants could access the survey (to confirm their current professional status and belonging to the target population). Finally, a closing question regarding facilitators to ESUR guidelines dissemination was presented after completing the last section. Overall, 16/24 were Likert scale questions, 7/24 were multiple choice questions and there was a single free-text question. All questions were marked as mandatory, so that only fully completed questionnaires could be received. Anonymity was ensured throughout the entire data collection process. A copy of the original questionnaire can be found in the Additional file 1.

### Survey promotion and answers collection

An electronic flyer including the link to access and take the survey was created and disseminated using the official social network accounts of the ESUR–Junior Network. Similarly, members of the ESUR–Junior Network Committee used their own personal social media accounts and personal networks to increase survey visibility. Furthermore, to reach a broader audience, national scientific societies associated with the European Society of Radiology were contacted to request their support in forwarding the survey link to their members. The survey was online from April 4, 2022, and closed on May 30, 2022.

### Statistical analysis

Discrete variables are presented as count and percentages. The mode, median, and interquartile range were calculated. Respondents were divided into subgroups using 6 items retrieved from the questionnaire (i.e., continent of residency, guidelines consultations, guidelines full-text reads, number of MRI staging exams seen during residency, current professional status, and interest in a subspecialty) to perform a subgroup analysis using the Kruskal–Wallis rank sum test and the Wilcoxon rank sum test with continuity correction for pairwise comparisons, when appropriate. A *p* value < 0.05 was considered statistically significant. All statistical tests were performed using R [9].

## Results

### Descriptive statistics

**Fig. 1:**
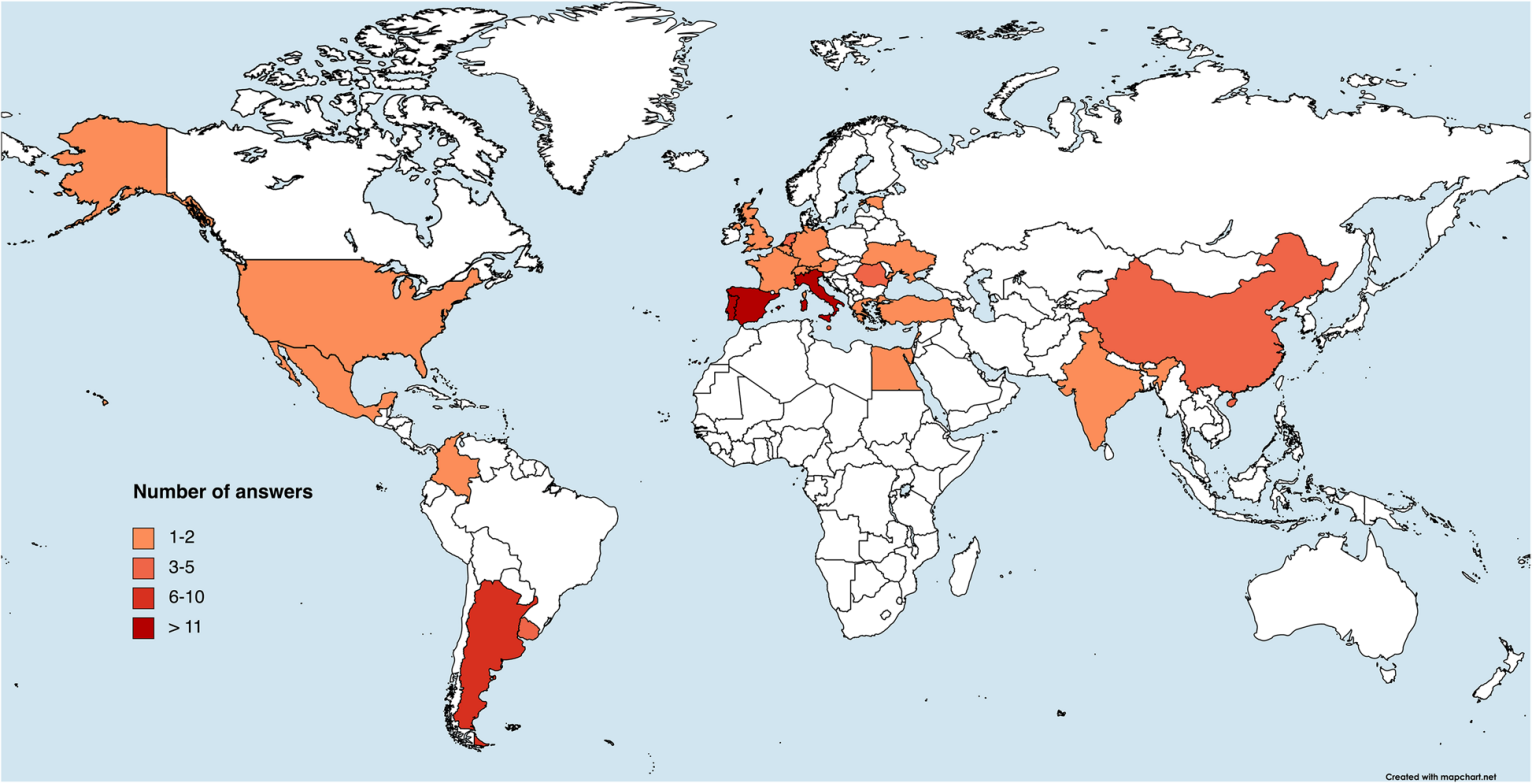
World heatmap showing respondent density and distribution

**Fig. 2:**
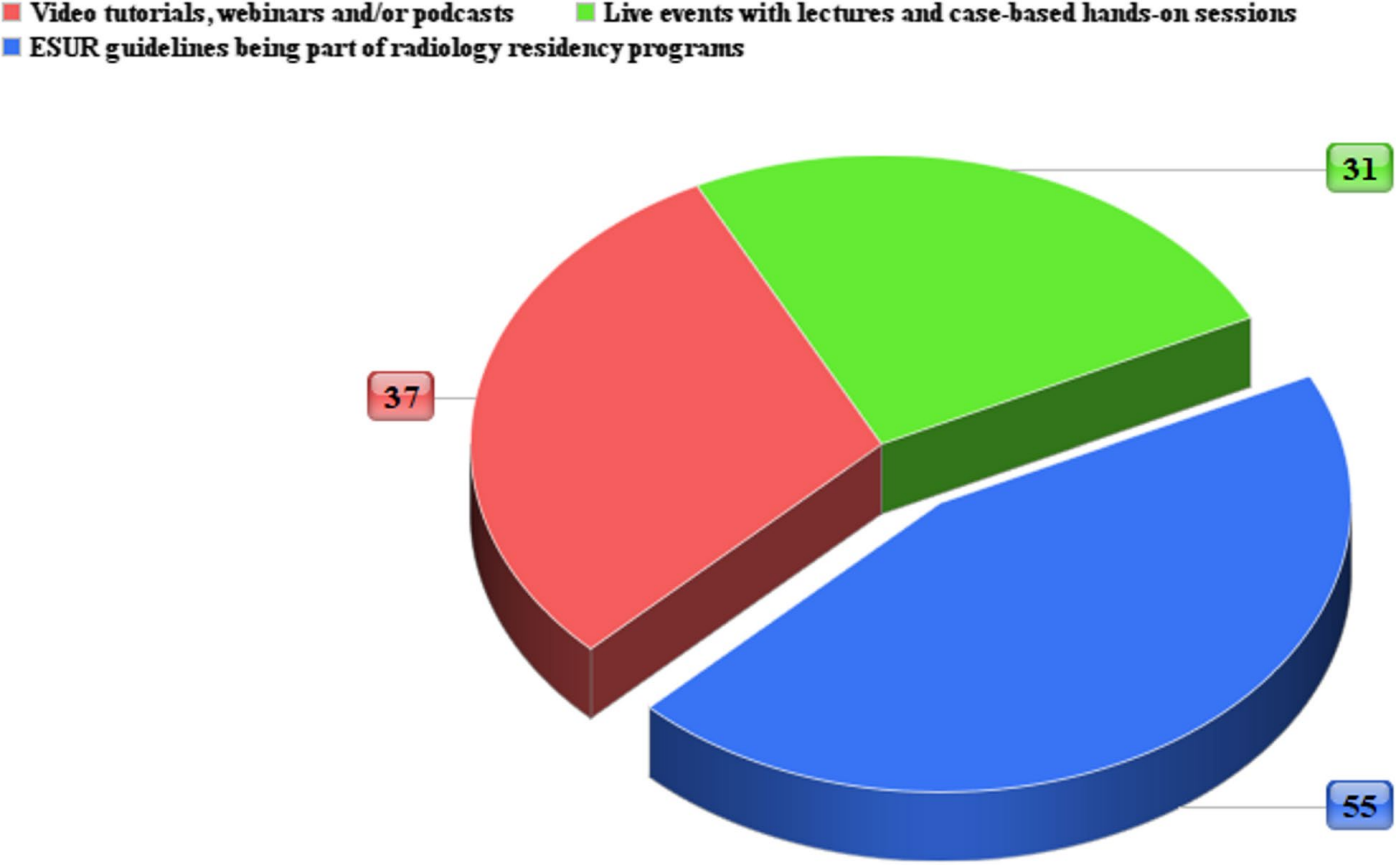
Pie chart showing answers (in percentages) to the question on preferred strategies to increase the dissemination of the guidelines

In total, 118 respondents completed the survey, with 94/118 (80%) being from the European continent. The geographical distributions of participants can be visualized in Fig. 1. Data collected from the answers received in the four questionnaire sections are reported in Tables 1, 2, 3, and 4. Briefly, 73/118 (62%) were last year radiology residents, 88/118 (75%) had seen at least 10 endometrial cancer MRI staging scans during residency, and 46/118 (39%) declared to have a special interest for urogenital radiology as a subspecialty. On a scale from 1 to 5, the perceived confidence with EC-ESUR guidelines content was reported as being ≤ 3 by 64/118 (54%) of participants, and 68/118 (58%) of them stated that the guidelines were not part of their residency teaching programs (e.g., not mentioned as a useful read by mentors, not presented at lectures for residents). A minority of respondents (29/118, 25%) indicated that referring physicians have mentioned the EC-ESUR guidelines during their residency (e.g., during multidisciplinary meetings). Almost one third (32/118, 27%) stated that they had never even consulted the guidelines. As for the final question, the results are shown in Fig. 2, with more than half respondents (55%) indicating that the inclusion of EC-ESUR guidelines in radiology residents’ teaching programs would be a facilitator to their dissemination among young radiologists.

**Table 1:** Background information collected from survey respondents

**Status**	Radiology residents *n* = 73 (62%)	Early carrier radiologists *n* = 45 (38%)		
**Gender**	Female *n* = 76 (64%)	Male *n* = 42 (36%)		
**Number of MRI scans for endometrial cancer staging during residency**	Less than 10 *n* = 30 (25%)	Between 10 and 30 *n* = 48 (41%)	Between 30 and 60 *n* = 26 (22%)	More than 60 *n* = 14 (12%)
**Interest in a subspecialty**	No (generalist) *n* = 32 (27%)	Yes (urogenital radiology) *n* = 46 (39%)	Yes (other) *n* = 40 (34%)

**Table 2:** Data collected in the general section of the questionnaire, with Likert scale questions in A and multiple-choice questions in B

	**1** **(not at all/strongly disagree)**	**2**	**3**	**4**	**5** **(completely/strongly agree)**	**Median (IQR)**	**Mode**
**Perceived confidence with ESUR guidelines on MRI endometrial cancer staging**	22 (18%)	14 (12%)	28 (24%)	41 (35%)	13 (11%)	3 (2)	4
**Have the guidelines been part of your residency program?**	27 (23%)	24 (20%)	17 (14%)	27 (23%)	23 (20%)	3 (2)	1
**Have the guidelines been part of your extracurricular professional growth?**	25 (21%)	18 (16%)	27 (23%)	26 (22%)	22 (18%)	3 (2)	3
**Referring physicians requested or mentioned the use of the guidelines at my institution**	32 (27%)	28 (24%)	28 (24%)	19 (16%)	10 (9%)	2 (2)	1

**Table 3:** Data collected in the section of the questionnaire focused on the acquisition protocol

	**1** **(not at all/strongly disagree)**	**2**	**3**	**4**	**5** **(completely/strongly agree)**	**Median (IQR)**	**Mode**
**Would you feel confident in supervising the acquisition of an MRI scan for endometrial cancer staging?**	16 (13%)	15 (13%)	28 (24%)	39 (33%)	20 (17%)	3.5 (2)	4
**Are T2W images on sagittal and axial oblique plane mandatory?**	1 (1%)	4 (3%)	9 (8%)	17 (14%)	87 (74%)	5 (1)	5
**Are fat-suppressed T2W images important?**	29 (25%)	19 (16%)	28 (24%)	20 (17%)	22 (18%)	3 (2)	1
**May IV administration be omitted in strictly selected cases?**	11 (10%)	24 (21%)	22 (18%)	30 (25%)	31 (26%)	4 (3)	5
**The use of DWI is not recommended**	63 (53%)	25 (21%)	13 (11%)	8 (7%)	9 (8%)	1 (1.75)	1
**Lymph node assessment: large FOV axial T2W is mandatory while DWI is optional**	11 (10%)	6 (5%)	24 (21%)	34 (28%)	43 (36%)	4 (2)	5

**Table 4:** Data collected in the section of the questionnaire focused on interpretation and reporting, with Likert scale questions in A and multiple-choice questions in B

	**1** **(not at all/strongly disagree)**	**2**	**3**	**4**	**5** **(completely/strongly agree)**	**Median (IQR)**	**Mode**
**Would you feel confident in interpreting and reporting an MRI scan for endometrial cancer staging?**	7 (6%)	21 (17%)	31 (26%)	48 (41%)	11 (10%)	3.5 (1)	4
**During my residency, I have familiarized with the deep myometrial invasion measurement strategy described in the guidelines**	23 (20%)	21 (17%)	27 (23%)	35 (30%)	12 (10%)	3 (2)	4
**How do you feel familiar with imaging pitfalls in endometrial cancer MRI staging?**	16 (14%)	31 (26%)	34 (28%)	28 (24%)	9 (8%)	3 (2)	3
**During my residency, I have familiarized with the structured report template proposed in the guidelines**	26 (10%)	23 (21%)	36 (18%)	22 (25%)	11 (26%)	3 (2)	3

### Subgroup analyses

The complete results from the subgroup analysis can be found in the Additional file 2. In summary, no statistically significant differences in terms of answers distribution were found between last year radiology residents and young radiologists in their first year of professional career.

Overall, physicians with a special interest in urogenital radiology showed a statistically significant higher perceived confidence with guidelines content, endometrial cancer MRI staging acquisition protocol as well as interpretation and reporting. No differences were found regarding the inclusion of EC-ESUR guidelines in residency programs and urogenital radiology sub-specialists were more likely to have familiarized with the guidelines independently during extracurricular activities (e.g., attending a webinar).

Few significant differences were found between the distribution of answers based on geographical location of residency (Europe vs other continents). Specifically, respondents from Europe were more inclined to consider the possibility of omitting contrast agent administration in carefully selected patients while participants from continents other than Europe reported a greater perceived confidence in the acquisition protocol. EC-ESUR guidelines were found more likely to be mentioned by referring physicians outside Europe.

Higher numbers of endometrial cancer MRI staging exams seen during residency were generally paired to higher confidence in EC-ESUR guidelines content, including pitfalls and structured report template, as well as perceived confidence in the ability to supervise scan acquisition and to interpret and report those exams. Similarly, those who consulted or read entirely the full-text of the EC-ESUR guidelines reported an overall greater degree of confidence with protocol acquisition as well as interpretation and reporting.

## Discussion

The results of this survey suggest that the degree of dissemination of EC-ESUR guidelines is lower than desirable among young radiologists. While in line with the findings of a 2019 survey on female imaging ESUR guidelines, the present evidence deepens our understanding of the issue [8]. Indeed, the present study was targeted on a very narrow window in the professional life of radiologists, showing that a major cause of the low adoption of EC-ESUR guidelines is their heterogeneous inclusion as teaching material in radiology residency programs. Considering that the guidelines have been published in mid-2018, the results of the 2019 survey might have been partly justified by the limited amount of time available to include the document in the teaching programs. However, if this was the case, a positive trend over time should have been observed. Conversely, radiologists in their first year of professional career and last year radiology residents have equally reported a low presence of EC-ESUR guidelines in their training curricula. This advocates for a more direct intervention and underlines the need for efforts in guideline promotion. At least to a certain extent, this observation may apply to other imaging guidelines released by ESUR as well. While raising the awareness of radiologists (especially those involved in education) seems an obvious solution, disseminating the guidelines among clinicians and surgeons might also be beneficial. Indeed, few respondents reported that referring physicians were requesting or mentioning the use of the guidelines. However, if convinced of their value, referring physicians could contribute to a more widespread adoption, activating a positive feedback circuit which might be beneficial in terms of homogenizing and increasing radiology report quality.

Due to the importance of endometrial cancer MRI staging, to the prevalence of the disease, and to the shortage of subspecialized radiologists, generalist radiologists are likely to face the challenge of accurately performing and assessing this type of scan. Unfortunately, it has been found that abdominal imaging studies (and endometrial cancer MRI in particular) from non-tertiary hospitals have heterogeneous quality, and second reads by subspecialized radiologists lead to better patient management [10, 11]. However, tumor board meetings generate a conspicuous workload for subspecialized radiologists and it could be speculated that a more widespread adoption of EC-ESUR guidelines might increase the overall quality of endometrial cancer MRI staging scans and reports, possibly reducing the need for second reads and leading to better patient care, although the present survey alone cannot confirm this hypothesis [12, 13].

It is also interesting to note that 51% of respondents reported a good perceived ability in interpreting and reporting endometrial cancer MRI staging exams; however, only 34% of participants reported to have seen more than 30 such exams during their residency. It could be therefore speculated that the EC-ESUR guidelines might be filling this apparent confidence gap, a theory that would be in line with what was found with the subgroup analyses regarding guideline consultations and full-text reads. Taken together, these findings support the educational value of the guidelines even outside the context of proper lectures and training (although these should ideally be paired).

The data collected in the protocol acquisition section deserve to be discussed. Indeed, encouraging answers were provided for the items regarding the importance of axial-oblique T2-weighted images and diffusion-weighted imaging. These findings indicate that despite the relatively low dissemination of the EC-ESUR guidelines, some crucial points in the acquisition protocols are well-recognized by young radiologists. Such a result could be at least partly explained by the fact that the participants have seen endometrial cancer MRI staging exams in academic institutions during their training and have retained the information regarding the most valuable sequences. Additionally, there is the possibility that radiology residents are being referred to other imaging guidelines (e.g., local or even institutional) with a reasonable agreement on EC-ESUR guidelines acquisition protocol recommendation. On the other hand, the answer distribution for the question on T2-weighted fat-suppressed images seems to suggest uncertainty among respondents regarding its role. EC-ESUR guidelines clearly state that T2-weighted fat-suppressed images are not recommended but it is possible that in non-subspecialized settings more sequences than necessary might be acquired. Similarly, answers on contrast agent administration were heterogeneous, and this might be related to the need for direct radiological supervision to consider the possibility of abbreviated unenhanced protocols in strictly selected cases, which might not always be feasible.

Regarding the structured report and the methodology for deep myometrial invasion proposed in the EC-ESUR guidelines, most respondents reported a poor or uncertain familiarity (respectively 85/118, 72% and 81/118, 69%). This finding is in line with the relatively low dissemination of the guidelines and also suggests that structured reporting and standardized myometrial invasion measurement are not routinely employed in several academic institutions.

Interestingly, among the strategies to increase EC-ESUR guidelines dissemination, their formal introduction into the residency program received the most preferences. Since they are European guidelines endorsed by the corresponding scientific society, a direct action to promote their formal adoption involving residency program’s heads is recommended and appears feasible. While it might be more challenging to increase their visibility in other continents, Europe could represent a good starting point, and with data demonstrating the advantages of greater dissemination in radiology residency programs, it might become easier to further promote EC-ESUR guidelines adoption. It should not be neglected that many respondents indicated alternative approaches (e.g., webinars or live courses) as valuable alternatives in aiding young radiologists to familiarize with the guidelines. Indeed, it can be speculated that seminars (live or digital) from leading experts in the field would increase the visibility of the EC-ESUR guidelines and it is intuitive that such initiatives could complement their adoption in residency programs. Henceforth, it appears that combining multiple active strategies for guidelines dissemination might be the best approach to reach a more widespread audience.

This study suffers from some limitations that should be acknowledged. Firstly, results are likely influenced by sampling bias, due to the high percentage of young radiologists with a particular interest in urogenital radiology being more likely to participate in the survey. Sampling bias is a common issue in surveys and tends to increase when a subgroup is targeted as the population of interest [14]. However, in our case, the sampling bias might have led to overoptimistic results regarding the widespread adoption of the guidelines, and this is not what emerged from the data. While this is not necessarily proof of the lack of such bias, it is likely that the distribution of the guidelines among our target population might be even lower. Secondly, the sample size is relatively small, and this might have especially biased the results from the geographical location analysis due to the skewness toward European responses. Furthermore, due to the promotion strategy of this survey, it was not possible to calculate the response rate. However, the overall number of respondents should be evaluated considering the objective was to collect a representative sample of young radiologists at the end of their training course. Additionally, statistically significant differences have emerged, suggesting sufficient statistical power. Finally, the possibility that imaging guidelines other than the EC-ESUR are adopted was not formally evaluated. However, to the best of our knowledge, we were not able to find imaging guidelines on the topic published in peer-reviewed international scientific journals, thus making the hypothesis of “competing guidelines” limiting the diffusion of EC-ESUR guidelines unlikely.

In conclusion, at 4 years after publication, the EC-ESUR guidelines are heterogeneously present in the training course of young radiologists, despite evidence of a valuable educational potential. The findings might similarly apply to other imaging guidelines as well. Active strategies to promote their dissemination and adoption are required.

### Supplementary Information

**Additional file 1.** Questionnaire.

**Additional file 2.** Results from the subgroup analysis.

## Data Availability

The datasets used and/or analyzed during the current study are available from the corresponding author on reasonable request.

## Abbreviations

ESUR: European Society of Urogenital Radiology
EC-ESUR guidelines: ESUR endometrial cancer MRI staging guidelines
