# Supplementary material for: Dissemination of endometrial cancer MRI staging guidelines among young radiologists: an ESUR Junior Network survey
Source: Insights Imaging. 2023 Sep 4;14:143. doi: 10.1186/s13244-023-01491-w (PMC10477141; doi:10.1186/s13244-023-01491-w)
Supplement: Supplementary file 1 — Additional file 1. Questionnaire. [file 13244_2023_1491_MOESM1_ESM.pdf]

Assessing ESUR Guidelines Educational Impact: Endometrial Cancer MRI Staging

In July 2018, the European Society of Urogenital Radiology (ESUR) published on European Radiology the Updated Guidelines for Endometrial Cancer MRI Staging (DOI: 10.1007/s00330-018-5515-y). With the present questionnarie, we aim to explore the educational impact of these guidelines in the training course of young radiologists.

The Questionnaire is organized into 4 sections (Background, General, Acquisition Protocol, Interpretation and Reporting) with no more than 6 questions each, plus a final question. Completing this survey will require approximately 5 minutes.

All data collected in this survey will be analyzed anonymously for research purposes. Please, keep in mind that this is NOT an exam and you should REFRAIN from consulting the Guidelines document before answering the questions. We are kindly asking for your sincere responses.

Before getting started, you will need to confirm your status (either last year radiology residents or radiologists within one year after certification are invited to participate).

Please, note that your answers will only be recorded after all sections have been thoroughly completed (a message will appear to confirm that your responses have been recorded).

We greatly appreciate your participation and wish to thank you in advance for your precious contribution.
Sincerely, the ESUR Junior Network

* Required

1. Please, confirm your status: *

Mark only one oval.

- ◯ Last year radiology resident
- ◯ Radiologist (within one year after certification)

BACKGROUND

Tell us something about you!

2. What is your gender? *

Mark only one oval.

- Male
- Female
- Other
- Prefer not to say

3. Please, indicate the country of your residency program: *

____

4. During your residency, how many MRI scans performed for endometrial cancer staging have you seen (this includes acquisition, interpretation and reporting, coherently with the resident status)? *

Mark only one oval.

- Less than 10
- Between 10 and 30
- Between 30 and 60
- More than 60

5. Have you grown a particular interest for a radiology sub-specialty during your residency? (If other, please specify) *

Mark only one oval.

- No (generalist radiologist)
- Yes (urogenital radiology)
- Other: _______________________________

GENERAL

In this section, we will explore your overall familiarity with the ESUR Guidelines on endometrial cancer MRI staging.

6. On a scale from 1 to 5, how do you feel confident with the ESUR Guidelines on endometrial cancer MRI staging? *

Mark only one oval.

	1	2	3	4	5	
Not at all	◯	◯	◯	◯	◯	Completely

7. On a scale from 1 to 5, to which extent do you agree with the following statement: "The ESUR Guidelines on endometrial cancer MRI staging have been part of my formal training during residency (e.g., mentioned/illustrated during lectures, consulted during MRI acquisition/interpretation/reporting, suggested as useful readings by the tutor)"? *

Mark only one oval.

	1	2	3	4	5	
Strongly disagree	◯	◯	◯	◯	◯	Strongly agree

8. On a scale from 1 to 5, to which extent do you agree with the following statement: "The ESUR Guidelines on endometrial cancer MRI staging have been part of my extracurricular professional growth (e.g., you found the paper and read it on your own, you participated in a webinar illustrating them, journal club activities, scientific meetings)"? *

Mark only one oval.

	1	2	3	4	5	
Strongly disagree	◯	◯	◯	◯	◯	Strongly agree

9. Have you ever consulted the original document (DOI: 10.1007/s00330-018-5515-y)? *

Mark only one oval.

◯ Never
◯ Once
◯ More than once

10. Have you ever read the entire original document (DOI: 10.1007/s00330-018-5515-y)? *

Mark only one oval.

- Never
- Once
- More than once

11. "On a scale from 1 to 5, to which extent do you agree with the following statement: "The ESUR Guidelines on endometrial cancer MRI staging have been either mentioned or their use requested by referring physicians in my institution (e.g., gynaecologists or oncologists, during multidisciplinary meetings or imaging re-evaluation)"?

Mark only one oval.

	1	2	3	4	5	
Strongly Disagree						Strongly Agree

ACQUISITION PROTOCOL

In this section, we will explore your overall familiarity with the MRI acquisition protocol recommended by ESUR for endometrial cancer staging.

12. On a scale from 1 to 5, how would you feel confident in supervising the MRI acquisition protocol for endometrial cancer staging? *

Mark only one oval.

	1	2	3	4	5	
Not at all						Completely

13. On a scale from 1 to 5, to which extent do you agree with the following statement: "Sagittal and axial oblique (perpendicular to endometrial cavity) two-dimensional T2W sequences through the uterus are mandatory to stage endometrial cancer."? *

Mark only one oval.

	1	2	3	4	5	
Strongly disagree	◯	◯	◯	◯	◯	Strongly agree

14. On a scale from 1 to 5, to which extent do you agree with the following statement: "Fat suppressed T2W sequences of the pelvis are an important part of the MRI protocol for endometrial cancer staging"? *

Mark only one oval.

	1	2	3	4	5	
Strongly disagree	◯	◯	◯	◯	◯	Strongly agree

15. On a scale from 1 to 5, to which extent do you agree with the following statement: "IV contrast administration may be omitted for endometrial cancer stanging in strictly selected cases and with the direct radiologist supervision"? *

Mark only one oval.

	1	2	3	4	5	
Strongly disagree	◯	◯	◯	◯	◯	Strongly agree

16. On a scale from 1 to 5, to which extent do you agree with the following statement: "the use of DWI is not recommended for endometrial cancer staging"? *

Mark only one oval.

	1	2	3	4	5	
Strongly disagree	◯	◯	◯	◯	◯	Strongly agree

17. On a scale from 1 to 5, to which extent do you agree with the following statement: "for lymph node assessment, axial T2W from the renal hila to the pubic symphysis is mandatory while axial DWI should be considered in selected patients"? *

Mark only one oval.

	1	2	3	4	5	
Strongly disagree	◯	◯	◯	◯	◯	Strongly agree

INTERPRETATION AND REPORTING

In this section, we will explore your overall familiarity with interpretation and reporting of MRI findings in endometrial cancer staging.

18. On a scale from 1 to 5, how would you feel confident in interpreting and reporting an MRI scan for endometrial cancer staging? *

Mark only one oval.

	1	2	3	4	5	
Not at all	◯	◯	◯	◯	◯	Completely

19. On a scale from 1 to 5, to which extent do you agree with the following statement: "During my residency, I have familiarized with the deep myometrial invasion measurement strategy described in the ESUR guidelines for endometrial cancer staging"? *

Mark only one oval.

	1	2	3	4	5	
Strongly disagree	◯	◯	◯	◯	◯	Strongly agree

20. On a scale from 1 to 5, how is it likely that you will use the deep myometrial invasion measurement strategy described in the ESUR guidelines for endometrial cancer staging? *

Mark only one oval.

- It is highly unlikely that I will be reporting MRI scans for endometrial cancer staging due to my professional profile
- Highly unlikely
- Unlikely
- Uncertain
- Likely
- Highly likely

21. On a scale from 1 to 5, how would you define your knowledge of the potential imaging pitfalls in MRI endometrial cancer staging? *

Mark only one oval.

	1	2	3	4	5	
Very poor						Very good

22. On a scale from 1 to 5, to which extent do you agree with the following statement: "During my residency, I have familiarized with the structured report template proposed for endometrial cancer staging in the ESUR guidelines"? *

Mark only one oval.

	1	2	3	4	5	
Strongly disagree						Strongly agree

23. On a scale from 1 to 5, how is it likely that you will use the structured report template recommended in the ESUR guidelines for endometrial cancer staging? *

Mark only one oval.

- It is highly unlikely that I will be reporting MRI scans for endometrial cancer staging due to my professional profile
- Highly unlikely
- Unlikely
- Uncertain
- Likely
- Highly likely

YOU ARE ALMOST DONE!

Final question

24. Which of the following would help the most radiology residents and early career radiologists in familiarizing with ESUR guidelines:

Mark only one oval.

- ESUR guidelines being part of radiology residency programs
- Live events with lectures and case-based hands-on sessions
- Video tutorials, webinars and/or podcasts
- Other: ____________________

This content is neither created nor endorsed by Google.

Google Forms
