# Supplementary material for: Dissemination of endometrial cancer MRI staging guidelines among young radiologists: an ESUR Junior Network survey
Source: Insights Imaging. 2023 Sep 4;14:143. doi: 10.1186/s13244-023-01491-w (PMC10477141; doi:10.1186/s13244-023-01491-w)
Supplement: Supplementary file 2 — Additional file 2. Results from the subgroup analysis. [file 13244_2023_1491_MOESM2_ESM.docx]

**Dissemination of Endometrial Cancer MRI Staging Guidelines Among Young Radiologists: An ESUR Junior Network Survey**

**ELECTRONIC SUPPLEMENTARY MATERIAL**

| *Results of the Kruskal-Wallis rank sum test using guidelines consultations as a variable* | | | | |
| --- | --- | --- | --- | --- |
| **Questionnaire item** | **More than once (n=51)** | **Once (n=35)** | **Never (n=32)** | ***p* value** |
| On a scale from 1 to 5, how do you feel confident with the ESUR Guidelines on endometrial cancer MRI staging? | 4 (1) | 3 (2.5) | 2 (2) | < 0.01* |
| On a scale from 1 to 5, to which extent do you agree with the following statement: "The ESUR Guidelines on endometrial cancer MRI staging have been part of my formal training during residency (e.g., mentioned/illustrated during lectures, consulted during MRI acquisition/interpretation/reporting, suggested as useful readings by the tutor)"? | 4 (2) | 3 (2) | 2 (2) | < 0.01* |
| On a scale from 1 to 5, to which extent do you agree with the following statement: "The ESUR Guidelines on endometrial cancer MRI staging have been part of my extracurricular professional growth (e.g., you found the paper and read it on your own, you participated in a webinar illustrating them, journal club activities, scientific meetings)"? | 4 (2) | 3 (2) | 1 (2) | < 0.01* |
| On a scale from 1 to 5, to which extent do you agree with the following statement: "The ESUR Guidelines on endometrial cancer MRI staging have been either mentioned or their use requested by referring physicians in my institution (e.g., gynaecologists or oncologists, during multidisciplinary meetings or imaging re-evaluation)"? | 3 (2) | 2 (3) | 2 (2) | 0.17 |
| On a scale from 1 to 5, how would you feel confident in supervising the MRI acquisition protocol for endometrial cancer staging? | 4 (2) | 3 (1) | 2 (2) | < 0.01* |
| On a scale from 1 to 5, to which extent do you agree with the following statement: "Sagittal and axial oblique (perpendicular to endometrial cavity) two-dimensional T2W sequences through the uterus are mandatory to stage endometrial cancer."? | 5 (0) | 5 (1) | 5 (1) | 0.03* |
| On a scale from 1 to 5, to which extent do you agree with the following statement: "Fat suppressed T2W sequences of the pelvis are an important part of the MRI protocol for endometrial cancer staging"? | 3 (3) | 3 (1.5) | 3 (2) | 0.15 |
| On a scale from 1 to 5, to which extent do you agree with the following statement: "IV contrast administration may be omitted for endometrial cancer stanging in strictly selected cases and with the direct radiologist supervision"? | 4 (3) | 3 (1.5) | 4 (3) | 0.71 |
| On a scale from 1 to 5, to which extent do you agree with the following statement: "the use of DWI is not recommended for endometrial cancer staging"? | 1 (1) | 2 (2) | 1 (2) | 0.31 |
| On a scale from 1 to 5, to which extent do you agree with the following statement: "for lymph node assessment, axial T2W from the renal hila to the pubic symphysis is mandatory while axial DWI should be considered in selected patients"? | 4 (2) | 4 (2) | 4 (2) | 0.97 |
| On a scale from 1 to 5, how would you feel confident in interpreting and reporting an MRI scan for endometrial cancer staging? | 4 (1) | 3 (1.5) | 2 (2) | < 0.01* |
| On a scale from 1 to 5, to which extent do you agree with the following statement: "During my residency, I have familiarized with the deep myometrial invasion measurement strategy described in the ESUR guidelines for endometrial cancer staging"? | 4 (1) | 3 (1.5) | 1.5 (2) | < 0.01* |
| On a scale from 1 to 5, how would you define your knowledge of the potential imaging pitfalls in MRI endometrial cancer staging? | 3 (1) | 3 (1) | 2 (2) | < 0.01* |
| On a scale from 1 to 5, to which extent do you agree with the following statement: "During my residency, I have familiarized with the structured report template proposed for endometrial cancer staging in the ESUR guidelines"? | 3 (1.5) | 3 (2) | 1.5 (2) | < 0.01* |
| On a scale from 1 to 5, how is it likely that you will use the deep myometrial invasion measurement strategy described in the ESUR guidelines for endometrial cancer staging?^*^ | 4 (1) | 4 (0) | 3.5 (1.25) | 0.03* |
| On a scale from 1 to 5, how is it likely that you will use the structured report template recommended in the ESUR guidelines for endometrial cancer staging?^§^ | 4 (2) | 4 (1) | 4 (1) | 0.71 |

* For this item, More than once (n=48) vs Once (n=20) vs Never (n=27) due to the exclusion of those answering that they were unlikely to be reporting such MRI exams at all.

§ For this item, More than once (n=49) vs Once (n=22) vs Never (n=30) due to the exclusion of those answering that they were unlikely to be reporting such MRI exams at all.

Data presented as median of Likert scale answers with interquartile range in parenthesis

**Results of the pairwise comparisons using Wilcoxon rank sum test with continuity correction (per item, when appropriate)**

1. On a scale from 1 to 5, how do you feel confident with the ESUR Guidelines on endometrial cancer MRI staging?

|  | **More than once** | **Never** |
| --- | --- | --- |
| **Never** | < 0.01* | # |
| **Once** | < 0.01* | 0.33 |

1. On a scale from 1 to 5, to which extent do you agree with the following statement: "The ESUR Guidelines on endometrial cancer MRI staging have been part of my formal training during residency (e.g., mentioned/illustrated during lectures, consulted during MRI acquisition/interpretation/reporting, suggested as useful readings by the tutor)"?

|  | **More than once** | **Never** |
| --- | --- | --- |
| **Never** | < 0.01* | # |
| **Once** | 0.01* | 0.02* |

1. On a scale from 1 to 5, to which extent do you agree with the following statement: "The ESUR Guidelines on endometrial cancer MRI staging have been part of my extracurricular professional growth (e.g., you found the paper and read it on your own, you participated in a webinar illustrating them, journal club activities, scientific meetings)"?

|  | **More than once** | **Never** |
| --- | --- | --- |
| **Never** | < 0.01* | # |
| **Once** | 0.02* | < 0.01* |

1. On a scale from 1 to 5, how would you feel confident in supervising the MRI acquisition protocol for endometrial cancer staging?

|  | **More than once** | **Never** |
| --- | --- | --- |
| **Never** | < 0.01* | # |
| **Once** | < 0.01* | < 0.01* |

1. On a scale from 1 to 5, to which extent do you agree with the following statement: "Sagittal and axial oblique (perpendicular to endometrial cavity) two-dimensional T2W sequences through the uterus are mandatory to stage endometrial cancer."?

|  | **More than once** | **Never** |
| --- | --- | --- |
| **Never** | 0.047* | # |
| **Once** | 0.04* | 0.79 |

1. On a scale from 1 to 5, how would you feel confident in interpreting and reporting an MRI scan for endometrial cancer staging?

|  | **More than once** | **Never** |
| --- | --- | --- |
| **Never** | < 0.01* | # |
| **Once** | < 0.01* | 0.05 |

1. On a scale from 1 to 5, to which extent do you agree with the following statement: "During my residency, I have familiarized with the deep myometrial invasion measurement strategy described in the ESUR guidelines for endometrial cancer staging"?

|  | **More than once** | **Never** |
| --- | --- | --- |
| **Never** | < 0.01* | # |
| **Once** | < 0.01* | < 0.01* |

1. On a scale from 1 to 5, how would you define your knowledge of the potential imaging pitfalls in MRI endometrial cancer staging?

|  | **More than once** | **Never** |
| --- | --- | --- |
| **Never** | < 0.01* | # |
| **Once** | < 0.01* | 0.05 |

1. On a scale from 1 to 5, to which extent do you agree with the following statement: "During my residency, I have familiarized with the structured report template proposed for endometrial cancer staging in the ESUR guidelines"?

|  | **More than once** | **Never** |
| --- | --- | --- |
| **Never** | < 0.01* | # |
| **Once** | 0.20 | < 0.01* |

1. On a scale from 1 to 5, how is it likely that you will use the deep myometrial invasion measurement strategy described in the ESUR guidelines for endometrial cancer staging?

|  | **More than once** | **Never** |
| --- | --- | --- |
| **Never** | 0.06 | # |
| **Once** | 0.16 | 0.21 |

| *Results of the Kruskal-Wallis rank sum test using geographic location as a variable* | | | |
| --- | --- | --- | --- |
| **Questionnaire item** | **EU (n=94)** | **Non-EU (n=24)** | ***p* value** |
| On a scale from 1 to 5, how do you feel confident with the ESUR Guidelines on endometrial cancer MRI staging? | 3 (2) | 4 (1) | 0.17 |
| On a scale from 1 to 5, to which extent do you agree with the following statement: "The ESUR Guidelines on endometrial cancer MRI staging have been part of my formal training during residency (e.g., mentioned/illustrated during lectures, consulted during MRI acquisition/interpretation/reporting, suggested as useful readings by the tutor)"? | 3 (2) | 3 (2.25) | 0.73 |
| On a scale from 1 to 5, to which extent do you agree with the following statement: "The ESUR Guidelines on endometrial cancer MRI staging have been part of my extracurricular professional growth (e.g., you found the paper and read it on your own, you participated in a webinar illustrating them, journal club activities, scientific meetings)"? | 3 (2) | 3 (2) | 0.80 |
| On a scale from 1 to 5, to which extent do you agree with the following statement: "The ESUR Guidelines on endometrial cancer MRI staging have been either mentioned or their use requested by referring physicians in my institution (e.g., gynaecologists or oncologists, during multidisciplinary meetings or imaging re-evaluation)"? | 2 (2) | 3 (2) | 0.008* |
| On a scale from 1 to 5, how would you feel confident in supervising the MRI acquisition protocol for endometrial cancer staging? | 3 (2) | 4 (1) | 0.005* |
| On a scale from 1 to 5, to which extent do you agree with the following statement: "Sagittal and axial oblique (perpendicular to endometrial cavity) two-dimensional T2W sequences through the uterus are mandatory to stage endometrial cancer."? | 5 (1) | 5 (0) | 0.19 |
| On a scale from 1 to 5, to which extent do you agree with the following statement: "Fat suppressed T2W sequences of the pelvis are an important part of the MRI protocol for endometrial cancer staging"? | 3 (2) | 3 (3.25) | 0.39 |
| On a scale from 1 to 5, to which extent do you agree with the following statement: "IV contrast administration may be omitted for endometrial cancer stanging in strictly selected cases and with the direct radiologist supervision"? | 4 (2) | 2.5 (2) | 0.011* |
| On a scale from 1 to 5, to which extent do you agree with the following statement: "the use of DWI is not recommended for endometrial cancer staging"? | 1 (2) | 1 (1) | 0.35 |
| On a scale from 1 to 5, to which extent do you agree with the following statement: "for lymph node assessment, axial T2W from the renal hila to the pubic symphysis is mandatory while axial DWI should be considered in selected patients"? | 4 (2) | 4 (2) | 0.99 |
| On a scale from 1 to 5, how would you feel confident in interpreting and reporting an MRI scan for endometrial cancer staging? | 4 (1) | 3 (2) | 0.28 |
| On a scale from 1 to 5, to which extent do you agree with the following statement: "During my residency, I have familiarized with the deep myometrial invasion measurement strategy described in the ESUR guidelines for endometrial cancer staging"? | 3 (1) | 4 (1) | 0.61 |
| On a scale from 1 to 5, how would you define your knowledge of the potential imaging pitfalls in MRI endometrial cancer staging? | 3 (2) | 3 (2) | 0.94 |
| On a scale from 1 to 5, to which extent do you agree with the following statement: "During my residency, I have familiarized with the structured report template proposed for endometrial cancer staging in the ESUR guidelines"? | 3 (1) | 3 (2.25) | 0.08 |
| On a scale from 1 to 5, how is it likely that you will use the deep myometrial invasion measurement strategy described in the ESUR guidelines for endometrial cancer staging?^*^ | 4 (1.75) | 4 (2) | 0.88 |
| On a scale from 1 to 5, how is it likely that you will use the structured report template recommended in the ESUR guidelines for endometrial cancer staging?^§^ | 4 (1) | 4 (2) | 0.31 |

EU: Europe

* For this item, EU (n=74) vs Non-EU (n=21) due to the exclusion of those answering that they were unlikely to be reporting such MRI exams at all.

§ For this item, EU (n=80) vs Non-EU (n=21) due to the exclusion of those answering that they were unlikely to be reporting such MRI exams at all.

Data presented as median of Likert scale answers with interquartile range in parenthesis

| *Results of the Kruskal-Wallis rank sum test using guidelines full-text readings as a variable* | | | | |
| --- | --- | --- | --- | --- |
| **Questionnaire item** | **More than once (n=27)** | **Never (n=54)** | **Once (n=37)** | ***p* value** |
| On a scale from 1 to 5, how do you feel confident with the ESUR Guidelines on endometrial cancer MRI staging? | 4 (0) | 2 (2) | 4 (1) | < 0.01* |
| On a scale from 1 to 5, to which extent do you agree with the following statement: "The ESUR Guidelines on endometrial cancer MRI staging have been part of my formal training during residency (e.g., mentioned/illustrated during lectures, consulted during MRI acquisition/interpretation/reporting, suggested as useful readings by the tutor)"? | 5 (2) | 2 (2) | 4 (1) | < 0.01* |
| On a scale from 1 to 5, to which extent do you agree with the following statement: "The ESUR Guidelines on endometrial cancer MRI staging have been part of my extracurricular professional growth (e.g., you found the paper and read it on your own, you participated in a webinar illustrating them, journal club activities, scientific meetings)"? | 4 (2) | 2 (2) | 4 (2) | < 0.01* |
| On a scale from 1 to 5, to which extent do you agree with the following statement: "The ESUR Guidelines on endometrial cancer MRI staging have been either mentioned or their use requested by referring physicians in my institution (e.g., gynaecologists or oncologists, during multidisciplinary meetings or imaging re-evaluation)"? | 3 (2) | 2 (2) | 3 (2) | < 0.01* |
| On a scale from 1 to 5, how would you feel confident in supervising the MRI acquisition protocol for endometrial cancer staging? | 4 (1) | 3 (2) | 4 (1) | < 0.01* |
| On a scale from 1 to 5, to which extent do you agree with the following statement: "Sagittal and axial oblique (perpendicular to endometrial cavity) two-dimensional T2W sequences through the uterus are mandatory to stage endometrial cancer."? | 5 (0) | 5 (1) | 5 (0) | 0.06 |
| On a scale from 1 to 5, to which extent do you agree with the following statement: "Fat suppressed T2W sequences of the pelvis are an important part of the MRI protocol for endometrial cancer staging"? | 2 (2) | 3 (2) | 3 (2) | 0.01* |
| On a scale from 1 to 5, to which extent do you agree with the following statement: "IV contrast administration may be omitted for endometrial cancer staging in strictly selected cases and with the direct radiologist supervision"? | 3 (2.5) | 3 (2) | 4 (2) | 0.04* |
| On a scale from 1 to 5, to which extent do you agree with the following statement: "the use of DWI is not recommended for endometrial cancer staging"? | 1 (0.5) | 2 (2) | 1 (2) | 0.10 |
| On a scale from 1 to 5, to which extent do you agree with the following statement: "for lymph node assessment, axial T2W from the renal hila to the pubic symphysis is mandatory while axial DWI should be considered in selected patients"? | 4 (3.5) | 4 (2) | 4 (2) | 0.22 |
| On a scale from 1 to 5, how would you feel confident in interpreting and reporting an MRI scan for endometrial cancer staging? | 4 (0) | 3 (1) | 4 (1) | < 0.01* |
| On a scale from 1 to 5, to which extent do you agree with the following statement: "During my residency, I have familiarized with the deep myometrial invasion measurement strategy described in the ESUR guidelines for endometrial cancer staging"? | 4 (1.5) | 2 (2) | 4 (1) | < 0.01* |
| On a scale from 1 to 5, how would you define your knowledge of the potential imaging pitfalls in MRI endometrial cancer staging? | 4 (1) | 2 (1.75) | 3 (1) | < 0.01* |
| On a scale from 1 to 5, to which extent do you agree with the following statement: "During my residency, I have familiarized with the structured report template proposed for endometrial cancer staging in the ESUR guidelines"? | 3 (1.5) | 2 (2) | 3 (1) | < 0.01* |
| On a scale from 1 to 5, how is it likely that you will use the deep myometrial invasion measurement strategy described in the ESUR guidelines for endometrial cancer staging?^*^ | 4 (1) | 4 (1) | 4 (1) | < 0.01* |
| On a scale from 1 to 5, how is it likely that you will use the structured report template recommended in the ESUR guidelines for endometrial cancer staging?^§^ | 4 (2) | 4 (1) | 4 (0.75) | 0.26 |

* For this item, More than once (n=26) vs Once (n=36) vs Never (n=33) due to the exclusion of those answering that they were unlikely to be reporting such MRI exams at all.

§ For this item, More than once (n=26) vs Once (n=41) vs Never (n=34) due to the exclusion of those answering that they were unlikely to be reporting such MRI exams at all.

Data presented as median of Likert scale answers with interquartile range in parenthesis

**Results of the pairwise comparisons using Wilcoxon rank sum test with continuity correction (per item, when appropriate)**

1. On a scale from 1 to 5, how do you feel confident with the ESUR Guidelines on endometrial cancer MRI staging?

|  | **More than once** | **Never** |
| --- | --- | --- |
| **Never** | < 0.01* | # |
| **Once** | 0.11 | < 0.01* |

1. On a scale from 1 to 5, to which extent do you agree with the following statement: "The ESUR Guidelines on endometrial cancer MRI staging have been part of my formal training during residency (e.g., mentioned/illustrated during lectures, consulted during MRI acquisition/interpretation/reporting, suggested as useful readings by the tutor)"?

|  | **More than once** | **Never** |
| --- | --- | --- |
| **Never** | < 0.01* | # |
| **Once** | 0.04* | < 0.01* |

1. On a scale from 1 to 5, to which extent do you agree with the following statement: "The ESUR Guidelines on endometrial cancer MRI staging have been part of my extracurricular professional growth (e.g., you found the paper and read it on your own, you participated in a webinar illustrating them, journal club activities, scientific meetings)"?

|  | **More than once** | **Never** |
| --- | --- | --- |
| **Never** | < 0.01* | # |
| **Once** | 0.90 | < 0.01* |

1. On a scale from 1 to 5, to which extent do you agree with the following statement: "The ESUR Guidelines on endometrial cancer MRI staging have been either mentioned or their use requested by referring physicians in my institution (e.g., gynaecologists or oncologists, during multidisciplinary meetings or imaging re-evaluation)"?

|  | **More than once** | **Never** |
| --- | --- | --- |
| **Never** | < 0.01* | # |
| **Once** | 0.94 | < 0.01* |

1. On a scale from 1 to 5, how would you feel confident in supervising the MRI acquisition protocol for endometrial cancer staging?

|  | **More than once** | **Never** |
| --- | --- | --- |
| **Never** | < 0.01* | # |
| **Once** | 0.02* | < 0.01* |

1. On a scale from 1 to 5, to which extent do you agree with the following statement: "Fat suppressed T2W sequences of the pelvis are an important part of the MRI protocol for endometrial cancer staging"?

|  | **More than once** | **Never** |
| --- | --- | --- |
| **Never** | 0.01* | # |
| **Once** | 0.03* | 0.98 |

1. On a scale from 1 to 5, to which extent do you agree with the following statement: "IV contrast administration may be omitted for endometrial cancer stanging in strictly selected cases and with the direct radiologist supervision"?

|  | **More than once** | **Never** |
| --- | --- | --- |
| **Never** | 0.51 | # |
| **Once** | 0.06 | 0.06 |

1. On a scale from 1 to 5, how would you feel confident in interpreting and reporting an MRI scan for endometrial cancer staging?

|  | **More than once** | **Never** |
| --- | --- | --- |
| **Never** | < 0.01* | # |
| **Once** | 0.38 | < 0.01* |

1. On a scale from 1 to 5, to which extent do you agree with the following statement: "During my residency, I have familiarized with the deep myometrial invasion measurement strategy described in the ESUR guidelines for endometrial cancer staging"?

|  | **More than once** | **Never** |
| --- | --- | --- |
| **Never** | < 0.01* | # |
| **Once** | 0.06 | < 0.01* |

1. On a scale from 1 to 5, how would you define your knowledge of the potential imaging pitfalls in MRI endometrial cancer staging?

|  | **More than once** | **Never** |
| --- | --- | --- |
| **Never** | < 0.01* | # |
| **Once** | 0.06 | 0.05 |

1. On a scale from 1 to 5, to which extent do you agree with the following statement: "During my residency, I have familiarized with the structured report template proposed for endometrial cancer staging in the ESUR guidelines"?

|  | **More than once** | **Never** |
| --- | --- | --- |
| **Never** | < 0.01* | # |
| **Once** | 0.85 | < 0.01* |

1. On a scale from 1 to 5, how is it likely that you will use the deep myometrial invasion measurement strategy described in the ESUR guidelines for endometrial cancer staging?

|  | **More than once** | **Never** |
| --- | --- | --- |
| **Never** | < 0.01* | # |
| **Once** | 0.54 | < 0.01* |

| *Results of the Kruskal-Wallis rank sum test using number of MRI exams witnessed/interpreted/reported as a variable* | | | | | |
| --- | --- | --- | --- | --- | --- |
| **Questionnaire item** | **Less than 10 (n=30)** | **Between 10 and 30 (n=48)** | **Between 30 and 60 (n=26)** | **More than 60 (n=14)** | ***p* value** |
| On a scale from 1 to 5, how do you feel confident with the ESUR Guidelines on endometrial cancer MRI staging? | 2 (2) | 3 (2) | 4 (1) | 4 (1) | < 0.01* |
| On a scale from 1 to 5, to which extent do you agree with the following statement: "The ESUR Guidelines on endometrial cancer MRI staging have been part of my formal training during residency (e.g., mentioned/illustrated during lectures, consulted during MRI acquisition/interpretation/reporting, suggested as useful readings by the tutor)"? | 1 (1) | 3.5 (2) | 4 (2) | 3.5 (1) | < 0.01* |
| On a scale from 1 to 5, to which extent do you agree with the following statement: "The ESUR Guidelines on endometrial cancer MRI staging have been part of my extracurricular professional growth (e.g., you found the paper and read it on your own, you participated in a webinar illustrating them, journal club activities, scientific meetings)"? | 3 (3) | 3 (2) | 3 (2.75) | 4 (0) | 0.03* |
| On a scale from 1 to 5, to which extent do you agree with the following statement: "The ESUR Guidelines on endometrial cancer MRI staging have been either mentioned or their use requested by referring physicians in my institution (e.g., gynaecologists or oncologists, during multidisciplinary meetings or imaging re-evaluation)"? | 2 (2) | 2 (2) | 3 (1.75) | 2 (1.75) | 0.02* |
| On a scale from 1 to 5, how would you feel confident in supervising the MRI acquisition protocol for endometrial cancer staging? | 2 (1.75) | 3 (1.25) | 4 (1) | 4 (1) | < 0.01* |
| On a scale from 1 to 5, to which extent do you agree with the following statement: "Sagittal and axial oblique (perpendicular to endometrial cavity) two-dimensional T2W sequences through the uterus are mandatory to stage endometrial cancer."? | 5 (1) | 5 (1) | 5 (1) | 5 (0) | 0.12 |
| On a scale from 1 to 5, to which extent do you agree with the following statement: "Fat suppressed T2W sequences of the pelvis are an important part of the MRI protocol for endometrial cancer staging"? | 3 (2) | 3 (3) | 3 (2) | 2.5 (2.75) | 0.72 |
| On a scale from 1 to 5, to which extent do you agree with the following statement: "IV contrast administration may be omitted for endometrial cancer staging in strictly selected cases and with the direct radiologist supervision"? | 4 (2) | 4 (2) | 3 (1.75) | 4 (2) | 0.41 |
| On a scale from 1 to 5, to which extent do you agree with the following statement: "the use of DWI is not recommended for endometrial cancer staging"? | 1 (1) | 1 (2) | 2 (1.75) | 1 (0.75) | 0.33 |
| On a scale from 1 to 5, to which extent do you agree with the following statement: "for lymph node assessment, axial T2W from the renal hila to the pubic symphysis is mandatory while axial DWI should be considered in selected patients"? | 5 (1) | 4 (2) | 4 (2) | 4 (2) | 0.10 |
| On a scale from 1 to 5, how would you feel confident in interpreting and reporting an MRI scan for endometrial cancer staging? | 2 (1) | 3 (1) | 4 (1) | 4 (0) | < 0.01* |
| On a scale from 1 to 5, to which extent do you agree with the following statement: "During my residency, I have familiarized with the deep myometrial invasion measurement strategy described in the ESUR guidelines for endometrial cancer staging"? | 1.5 (1) | 3 (2) | 4 (1) | 4 (1) | < 0.01* |
| On a scale from 1 to 5, how would you define your knowledge of the potential imaging pitfalls in MRI endometrial cancer staging? | 2 (2) | 3 (2) | 3 (1) | 4 (1) | < 0.01* |
| On a scale from 1 to 5, to which extent do you agree with the following statement: "During my residency, I have familiarized with the structured report template proposed for endometrial cancer staging in the ESUR guidelines"? | 2 (1.75) | 3 (2) | 3 (1) | 3 (1) | < 0.01* |
| On a scale from 1 to 5, how is it likely that you will use the deep myometrial invasion measurement strategy described in the ESUR guidelines for endometrial cancer staging?* | 4 (1.75) | 4 (2) | 4 (1) | 4 (1.75) | 0.61 |
| On a scale from 1 to 5, how is it likely that you will use the structured report template recommended in the ESUR guidelines for endometrial cancer staging?^§^ | 4 (1.25) | 4 (1) | 4 (2) | 4 (1) | 0.61 |

* For this item, Less than 10 (n=36) vs Between 10 and 30 (n=23) vs Between 30 and 60 (n=22) vs More than 60 (n=14) due to the exclusion of those answering that they were unlikely to be reporting such MRI exams at all.

§ For this item, Less than 10 (n=40) vs Between 10 and 30 (n=24) vs Between 30 and 60 (n=24) vs More than 60 (n=13) due to the exclusion of those answering that they were unlikely to be reporting such MRI exams at all.

Data presented as median of Likert scale answers with interquartile range in parenthesis

**Results of the pairwise comparisons using Wilcoxon rank sum test with continuity correction (per item, when appropriate)**

1. On a scale from 1 to 5, how do you feel confident with the ESUR Guidelines on endometrial cancer MRI staging?

|  | **Between 10 and 30** | **Between 30 and 60** | **Less than 10** |
| --- | --- | --- | --- |
| **Between 30 and 60** | 0.11 | # | # |
| **Less than 10** | 0.02* | < 0.01* | # |
| **More than 60** | 0.26 | 0.88 | 0.02* |

1. On a scale from 1 to 5, to which extent do you agree with the following statement: "The ESUR Guidelines on endometrial cancer MRI staging have been part of my formal training during residency (e.g., mentioned/illustrated during lectures, consulted during MRI acquisition/interpretation/reporting, suggested as useful readings by the tutor)"?

|  | **Between 10 and 30** | **Between 30 and 60** | **Less than 10** |
| --- | --- | --- | --- |
| **Between 30 and 60** | 0.21 | # | # |
| **Less than 10** | < 0.01* | < 0.01* | # |
| **More than 60** | 0.56 | 0.65 | < 0.01* |

1. On a scale from 1 to 5, to which extent do you agree with the following statement: "The ESUR Guidelines on endometrial cancer MRI staging have been part of my extracurricular professional growth (e.g., you found the paper and read it on your own, you participated in a webinar illustrating them, journal club activities, scientific meetings)"?

|  | **Between 10 and 30** | **Between 30 and 60** | **Less than 10** |
| --- | --- | --- | --- |
| **Between 30 and 60** | 0.38 | # | # |
| **Less than 10** | 0.47 | 0.21 | # |
| **More than 60** | 0.03* | 0.21 | 0.03* |

1. On a scale from 1 to 5, to which extent do you agree with the following statement: "The ESUR Guidelines on endometrial cancer MRI staging have been either mentioned or their use requested by referring physicians in my institution (e.g., gynaecologists or oncologists, during multidisciplinary meetings or imaging re-evaluation)"?

|  | **Between 10 and 30** | **Between 30 and 60** | **Less than 10** |
| --- | --- | --- | --- |
| **Between 30 and 60** | 0.05 | # | # |
| **Less than 10** | 0.50 | 0.01* | # |
| **More than 60** | 0.83 | 0.20 | 0.50 |

1. On a scale from 1 to 5, how would you feel confident in supervising the MRI acquisition protocol for endometrial cancer staging?

|  | **Between 10 and 30** | **Between 30 and 60** | **Less than 10** |
| --- | --- | --- | --- |
| **Between 30 and 60** | 0.04* | # | # |
| **Less than 10** | 0.02* | < 0.01* | # |
| **More than 60** | < 0.01* | 0.22 | < 0.01* |

1. On a scale from 1 to 5, how would you feel confident in interpreting and reporting an MRI scan for endometrial cancer staging?

|  | **Between 10 and 30** | **Between 30 and 60** | **Less than 10** |
| --- | --- | --- | --- |
| **Between 30 and 60** | 0.11 | # | # |
| **Less than 10** | < 0.01* | < 0.01* | # |
| **More than 60** | < 0.01* | 0.07 | < 0.01* |

1. On a scale from 1 to 5, to which extent do you agree with the following statement: "During my residency, I have familiarized with the deep myometrial invasion measurement strategy described in the ESUR guidelines for endometrial cancer staging"?

|  | **Between 10 and 30** | **Between 30 and 60** | **Less than 10** |
| --- | --- | --- | --- |
| **Between 30 and 60** | 0.02 | # | # |
| **Less than 10** | < 0.01* | < 0.01* | # |
| **More than 60** | 0.05 | 0.96 | < 0.01* |

1. On a scale from 1 to 5, how would you define your knowledge of the potential imaging pitfalls in MRI endometrial cancer staging?

|  | **Between 10 and 30** | **Between 30 and 60** | **Less than 10** |
| --- | --- | --- | --- |
| **Between 30 and 60** | 0.03* | # | # |
| **Less than 10** | 0.03* | < 0.01* | # |
| **More than 60** | 0.03* | 0.46 | < 0.01* |

1. On a scale from 1 to 5, to which extent do you agree with the following statement: "During my residency, I have familiarized with the structured report template proposed for endometrial cancer staging in the ESUR guidelines"?

|  | **Between 10 and 30** | **Between 30 and 60** | **Less than 10** |
| --- | --- | --- | --- |
| **Between 30 and 60** | 0.19 | # | # |
| **Less than 10** | < 0.01* | < 0.01* | # |
| **More than 60** | 0.19 | 0.82 | < 0.01* |

| *Results of the Kruskal-Wallis rank sum test using professional status as a variable* | | | |
| --- | --- | --- | --- |
| **Questionnaire item** | **Resident (n=73)** | **Radiologist (n=45)** | ***p* value** |
| On a scale from 1 to 5, how do you feel confident with the ESUR Guidelines on endometrial cancer MRI staging? | 3 (2) | 3 (2) | 0.62 |
| On a scale from 1 to 5, to which extent do you agree with the following statement: "The ESUR Guidelines on endometrial cancer MRI staging have been part of my formal training during residency (e.g., mentioned/illustrated during lectures, consulted during MRI acquisition/interpretation/reporting, suggested as useful readings by the tutor)"? | 3 (2) | 3 (2) | 0.99 |
| On a scale from 1 to 5, to which extent do you agree with the following statement: "The ESUR Guidelines on endometrial cancer MRI staging have been part of my extracurricular professional growth (e.g., you found the paper and read it on your own, you participated in a webinar illustrating them, journal club activities, scientific meetings)"? | 3 (2) | 3 (2) | 0.69 |
| On a scale from 1 to 5, to which extent do you agree with the following statement: "The ESUR Guidelines on endometrial cancer MRI staging have been either mentioned or their use requested by referring physicians in my institution (e.g., gynaecologists or oncologists, during multidisciplinary meetings or imaging re-evaluation)"? | 2 (2) | 2 (3) | 0.81 |
| On a scale from 1 to 5, how would you feel confident in supervising the MRI acquisition protocol for endometrial cancer staging? | 3 (1) | 4 (2) | 0.70 |
| On a scale from 1 to 5, to which extent do you agree with the following statement: "Sagittal and axial oblique (perpendicular to endometrial cavity) two-dimensional T2W sequences through the uterus are mandatory to stage endometrial cancer."? | 5 (0) | 5 (1) | 0.15 |
| On a scale from 1 to 5, to which extent do you agree with the following statement: "Fat suppressed T2W sequences of the pelvis are an important part of the MRI protocol for endometrial cancer staging"? | 3 (3) | 3 (2) | 0.28 |
| On a scale from 1 to 5, to which extent do you agree with the following statement: "IV contrast administration may be omitted for endometrial cancer stanging in strictly selected cases and with the direct radiologist supervision"? | 4 (3) | 3 (2) | 0.50 |
| On a scale from 1 to 5, to which extent do you agree with the following statement: "the use of DWI is not recommended for endometrial cancer staging"? | 1 (1) | 1 (2) | 0.69 |
| On a scale from 1 to 5, to which extent do you agree with the following statement: "for lymph node assessment, axial T2W from the renal hila to the pubic symphysis is mandatory while axial DWI should be considered in selected patients"? | 4 (2) | 4 (2) | 0.46 |
| On a scale from 1 to 5, how would you feel confident in interpreting and reporting an MRI scan for endometrial cancer staging? | 4 (1) | 3 (2) | 0.67 |
| On a scale from 1 to 5, to which extent do you agree with the following statement: "During my residency, I have familiarized with the deep myometrial invasion measurement strategy described in the ESUR guidelines for endometrial cancer staging"? | 3 (2) | 3 (2) | 0.82 |
| On a scale from 1 to 5, how would you define your knowledge of the potential imaging pitfalls in MRI endometrial cancer staging? | 3 (2) | 3 (2) | 0.66 |
| On a scale from 1 to 5, to which extent do you agree with the following statement: "During my residency, I have familiarized with the structured report template proposed for endometrial cancer staging in the ESUR guidelines"? | 3 (2) | 3 (3) | 0.47 |
| On a scale from 1 to 5, how is it likely that you will use the deep myometrial invasion measurement strategy described in the ESUR guidelines for endometrial cancer staging?^*^ | 4 (2) | 4 (1) | 0.93 |
| On a scale from 1 to 5, how is it likely that you will use the structured report template recommended in the ESUR guidelines for endometrial cancer staging?^§^ | 4 (1) | 4 (1.75) | 0.31 |

* For this item, Resident (n=60) vs Radiologist (n=35) due to the exclusion of those answering that they were unlikely to be reporting such MRI exams at all.

§ For this item, Resident (n=63) vs Radiologist (n=38) due to the exclusion of those answering that they were unlikely to be reporting such MRI exams at all.

Data presented as median of Likert scale answers with interquartile range in parenthesis

| *Results of the Kruskal-Wallis rank sum test using interest in urogenital radiology as a variable* | | | |
| --- | --- | --- | --- |
| **Questionnaire item** | **Generalist or other (n=72)** | **Urogenital (n=46)** | ***p* value** |
| On a scale from 1 to 5, how do you feel confident with the ESUR Guidelines on endometrial cancer MRI staging? | 3 (2) | 4 (1) | < 0.01* |
| On a scale from 1 to 5, to which extent do you agree with the following statement: "The ESUR Guidelines on endometrial cancer MRI staging have been part of my formal training during residency (e.g., mentioned/illustrated during lectures, consulted during MRI acquisition/interpretation/reporting, suggested as useful readings by the tutor)"? | 3 (2) | 3.5 (2.75) | 0.20 |
| On a scale from 1 to 5, to which extent do you agree with the following statement: "The ESUR Guidelines on endometrial cancer MRI staging have been part of my extracurricular professional growth (e.g., you found the paper and read it on your own, you participated in a webinar illustrating them, journal club activities, scientific meetings)"? | 3 (3) | 3.5 (3) | 0.02* |
| On a scale from 1 to 5, to which extent do you agree with the following statement: "The ESUR Guidelines on endometrial cancer MRI staging have been either mentioned or their use requested by referring physicians in my institution (e.g., gynaecologists or oncologists, during multidisciplinary meetings or imaging re-evaluation)"? | 2 (2) | 2 (3) | 0.93 |
| On a scale from 1 to 5, how would you feel confident in supervising the MRI acquisition protocol for endometrial cancer staging? | 3 (2) | 4 (2) | < 0.01* |
| On a scale from 1 to 5, to which extent do you agree with the following statement: "Sagittal and axial oblique (perpendicular to endometrial cavity) two-dimensional T2W sequences through the uterus are mandatory to stage endometrial cancer."? | 5 (1) | 5 (0) | 0.01* |
| On a scale from 1 to 5, to which extent do you agree with the following statement: "Fat suppressed T2W sequences of the pelvis are an important part of the MRI protocol for endometrial cancer staging"? | 3 (3) | 2 (2) | < 0.01* |
| On a scale from 1 to 5, to which extent do you agree with the following statement: "IV contrast administration may be omitted for endometrial cancer stanging in strictly selected cases and with the direct radiologist supervision"? | 3 (2.25) | 4 (3) | 0.51 |
| On a scale from 1 to 5, to which extent do you agree with the following statement: "the use of DWI is not recommended for endometrial cancer staging"? | 2 (2) | 1 (1) | < 0.01* |
| On a scale from 1 to 5, to which extent do you agree with the following statement: "for lymph node assessment, axial T2W from the renal hila to the pubic symphysis is mandatory while axial DWI should be considered in selected patients"? | 4 (2) | 4 (2) | 0.42 |
| On a scale from 1 to 5, how would you feel confident in interpreting and reporting an MRI scan for endometrial cancer staging? | 3 (2) | 4 (0.75) | < 0.01* |
| On a scale from 1 to 5, to which extent do you agree with the following statement: "During my residency, I have familiarized with the deep myometrial invasion measurement strategy described in the ESUR guidelines for endometrial cancer staging"? | 3 (1) | 4 (1) | < 0.01* |
| On a scale from 1 to 5, how would you define your knowledge of the potential imaging pitfalls in MRI endometrial cancer staging? | 2 (1) | 3.5 (1) | < 0.01* |
| On a scale from 1 to 5, to which extent do you agree with the following statement: "During my residency, I have familiarized with the structured report template proposed for endometrial cancer staging in the ESUR guidelines"? | 2 (2) | 3 (1) | < 0.01* |
| On a scale from 1 to 5, how is it likely that you will use the deep myometrial invasion measurement strategy described in the ESUR guidelines for endometrial cancer staging?^*^ | 4 (1) | 4 (1) | 0.44 |
| On a scale from 1 to 5, how is it likely that you will use the structured report template recommended in the ESUR guidelines for endometrial cancer staging?^§^ | 4 (1) | 4 (2) | 0.33 |

* For this item, Generalist or other (n=51) vs Urogenital (n=44) due to the exclusion of those answering that they were unlikely to be reporting such MRI exams at all.

§ For this item, Generalist or other (n=55) vs Urogenital (n=46) due to the exclusion of those answering that they were unlikely to be reporting such MRI exams at all.

Data presented as median of Likert scale answers with interquartile range in parenthesis
